# Jailed Balloon Technique Versus Jailed Wire Technique for Side Branch Ostium Protection in Bifurcation Lesions: Evidence from Three-dimensional Optical Coherence Tomography Analysis

**DOI:** 10.31083/j.rcm2508300

**Published:** 2024-08-21

**Authors:** JianGuo Cui, Xun Wu, QinHua Jin, YunDai Chen

**Affiliations:** ^1^School of Medicine, Nankai University, 300071 Tianjin, China; ^2^Department of Cardiology, The First Medical Center, Chinese PLA General Hospital, 100853 Beijing, China

**Keywords:** optical coherence tomography, jailed balloon technique, jailed wire technique, bifurcation lesion

## Abstract

**Background:**

There is controversy regarding the effectiveness the of jailed wire technique (JWT) and jailed balloon technique (JBT) in preserving the side branch (SB) during treatment. This study compares the protective effect of JBT versus JWT on the SB ostium area in coronary bifurcation lesions using three-dimensional optical coherence tomography (3D-OCT).

**Methods:**

We obtained data from coronary heart disease patients who received OCT-guided percutaneous coronary intervention (PCI) for bifurcation lesions. The SB protection strategies were divided into JWT and JBT, with the latter further subdivided into active JBT (A-JBT) and conventional JBT (C-JBT). The primary endpoint was the SB ostium area difference measured by 3D-OCT before and after PCI. Partial correlation analysis and propensity score matching (PSM) was used to mitigate confounding biases.

**Results:**

A total of 207 bifurcation lesions from 191 patients were analyzed, including 136 lesions treated with JWT and 71 lesions treated with JBT. The SB ostium area was significantly greater in the JBT group compared to the JWT group (0.41 ± 1.22 mm^2^ vs. –0.25 ± 1.40 mm^2^, *p* = 0.001). Following 1:1 PSM to adjust for 60 pairs, the difference between groups was not statistically significant (0.28 ± 1.06 mm^2^ vs. –0.02 ± 1.29 mm^2^, *p* = 0.165). Subgroup analysis revealed that A-JBT provided superior protection in both true (0.47 ± 1.22 mm^2^ vs. –0.10 ± 1.10 mm^2^, *p* = 0.011) and non-true bifurcation lesions (0.56 ± 1.43 mm^2^ vs. –0.38 ± 1.62 mm^2^, *p* = 0.030) over JWT, while C-JBT provided protection similar to JWT. A positive partial correlation was observed between the diameter of the jailed balloon and the increase in SB ostium area (r = 0.296, *p* = 0.013).

**Conclusions:**

Overall, A-JBT, but not C-JBT, provided better protection in bifurcation lesions compared to JWT. The larger diameter of the jailed balloon, rather than the application of higher pressure, enhanced the SB protection.

## 1. Introduction

Bifurcation lesions account for 20% of all percutaneous coronary interventions (PCI), with provisional stenting being the predominant strategy for treating de novo coronary bifurcation lesions [1, 2]. The process of stent placement in the main vessel (MV) may lead to plaque redistribution or carina shift, subsequently impacting the side branch (SB) [3]. This can escalate into SB complications ranging from ostial stenosis deterioration to complete SB occlusion, potentially resulting in perioperative myocardial infarction and adverse long-term outcomes [4, 5]. Previous studies have shown that SB ostial stenosis is an independent predictor of acute SB occlusion following MV stenting [6, 7]. Therefore, it is crucial to address SB ostium stenosis during bifurcation lesion interventions.

The jailed balloon technique (JBT) and jailed wire technique (JWT) are key strategies for protecting the SB and mitigating SB occlusion risk in complex bifurcation lesions [8, 9]. Despite their widespread use, comparative data on JBT and JWT is limited and has yielded mixed results [10, 11]. Studies indicate JWT was associated with fewer instances of SB occlusion following MV stenting in cases of severe stenosis at the SB or MV [10]. In contrast, the CIT-RESOLVE study showed that JBT provided superior SB protection compared to JWT [12], a finding contested by another research group [13]. This discrepancy underscores the need for further investigation to clarify the comparative efficacy of these techniques.

The evaluation of SB complications often relies on immediate angiographic results. However, accurately assessing SB ostial stenosis is challenging due to factors such as vessel overlap, angulations, stent struts obstructing the branch view, and image foreshortening [14]. These inherent limitations complicate the clarity of assessments.

Three-dimensional (3D) optical coherence tomography (OCT) has emerged as a sophisticated intravascular imaging tool, offering detailed insights into the coronary lumen and vessel wall. It facilitates an indirect yet precise assessment of the SB ostium area, overcoming the drawbacks of traditional imaging techniques [15, 16]. The effectiveness of 3D-OCT in accurately measuring the SB ostium area has been demonstrated, positioning it as a reliable alternative to direct OCT pullback examinations of the SB [15]. Given its potential, this imaging method is poised to significantly influence the interventional treatment of bifurcation lesions. To our knowledge, no studies have utilized 3D-OCT to assess the protective effect of JBT and JWT on the SB.

Therefore, the objective of this study was to evaluate the protective effects of JBT and JWT on the SB ostium using 3D-OCT. By employing this innovative approach, we anticipate the ability to provide more comprehensive data on the efficacy of JBT and JWT, thereby aiding in the clinical decision-making process.

## 2. Study and Methods

### 2.1 Populations

Between September 2019 and March 2022, we conducted a retrospective screening of the coronary artery OCT imaging database at a national high-volume tertiary referral center. The inclusion criteria were as follows: (ⅰ) high quality OCT pullbacks obtained from the main branch. (ⅱ) Availability of OCT pullback data both before and after PCI. (ⅲ) The presence of at least one side branch affected by stent deployment in the main vessel. (ⅳ) The side branch ostium being sufficiently large to allow for three-dimensional visualization. The exclusion criteria were as follows: (ⅰ) Poor image quality (e.g., incomplete flushing) or image artifacts (e.g., guidewire shadow) that impeded 3D rendering and visualization of the SB ostium. (ⅱ) Inability to match the OCT image of the SB with coronary angiography. Patients were then categorized into either the JWT or the JBT group based on the side branch protection strategy employed.

### 2.2 Coronary Angiography and Interventional Procedures

All procedures were performed by experienced interventional clinicians following standard techniques at our institution. The choice of intervention strategy was left to the discretion of the physician. Prior to the intervention, all patients received a loading dose of aspirin (300 mg) and either clopidogrel (600 mg) or ticagrelor (180 mg) at least 24 hours in advance. Additionally, all patients received unfractionated heparin at a rate of 100 mg/kg to achieve an activated clotting time of 250–350 seconds. After intracoronary injection of 100–200 µg nitroglycerin, coronary angiography was conducted via the radial or femoral approach.

### 2.3 Percutaneous Coronary Intervention Strategy

For the JWT procedure, coronary guidewires were placed distally in the MV and SB respectively. The MV lesions were routinely prepared, and the preparation of SB lesions was based on the operator’s discretion. The wire for SB protection was kept in place, and MV stents were deployed with a size optimized for the distal MV.

In the JBT procedure, the use of guidewires and the preparation of MV or SB lesions were similar to that of JWT. A jailed balloon with a diameter of 1.5 mm to 2.5 mm was advanced over the guide wire and positioned at the SB ostial site. The proximal protrusion of the balloon was adjusted to the MV by approximately 2 mm. The MV stents were deployed with a size optimized for the distal MV. Note that A-JBT refers to simultaneous dilation of the main branch stent and the SB jailed balloon, while C-JBT refers to dilation of the main branch stent without dilation of the SB jailed balloon. The dilation pressure of the jailed balloon was clustered on 4 atm and the diameter clustered on 2 mm. A low dilation pressure of the jailed balloon was defined as ≤4 atm, and a high dilation pressure was defined as >4 atm. A small diameter of the jailed balloon was defined as ≤2.0 mm, and a large diameter was defined as >2.0 mm.

### 2.4 Optical Coherence Tomography Image Acquisition and Analysis

The OCT image acquisition was conducted using two different systems: the commercially available C7-XRTM OCT intravascular image acquisition system (St Jude/LightLab Imaging, Inc., Westford, MA, USA) with a Dragonfly catheter (St Jude /LightLab Imaging, Inc., Westford, MA, USA) (N = 126, L = 132), and the CornarisTM system (Vivolight Corporation, Shenzhen, China) with a Pathfinder164 catheter (Vivolight Corporation, Shenzhen, China) (N = 65, L = 75). The OCT catheter was advanced over the guide wire and positioned at least 10 mm distal to the target lesion in the tested artery. Automated OCT pullback was performed at a speed of 20 mm/s while continuously injecting contrast medium (Iodixanol 370, Visipaque TM, GE HealthCare, Ireland) through the guiding catheter at a rate of 3–4 mL/s. The OCT images were analyzed offline by two experienced investigator (QHJ and JGC) who were blinded to the information. The analysis was performed according to a predefined standard operating procedure using available software (Vivolight Imaging Systems, Shenzhen, China). The SB ostium area was measured using a cut-plane analysis based on a 3D model [15]. Plaque types were classified based on criteria from previous studies, and were divided into fibrous plaques, lipid-rich plaques, and fibrocalcific plaques. A normal vessel wall was defined as having mild intimal hyperplasia or a typical three-layer structure of the intima, media, and adventitia [17].

### 2.5 Endpoints

The primary efficacy endpoint was the difference in SB ostium areas, while the safety endpoint was the quantification of SB protection procedure related complications. The area of the SB ostium was measured using Vivolight OCT software in a 3D model before and after single stenting of the MV. The SB ostium area difference was calculated as the post-PCI SB ostium area minus the pre-PCI SB ostium area. Complications related to JBT or JWT were defined as SB dissection, entrapment of guidewires, or entrapment of balloons.

### 2.6 Statistical Analysis

Continuous variables with a normal distribution were reported as mean ± standard deviation, while nonnormal variables were presented as the median ± interquartile range. To compare the differences between two independent groups with normal and nonnormal distributions, Student’s* t* tests and Mann–Whitney U tests were used, respectively. Categorical data were presented as numbers and percentages, and analyzed using either the chi-square test or Fisher’s exact test, as appropriate. Correlations between jailed balloon pressure, jailed balloon diameter, and SB ostium area change were evaluated using Spearman’s rank correlation coefficient and partial correlation analysis.

To mitigate confounding biases linked to an intention-to-treat analysis, propensity score matching (PSM) was applied. Factors influencing outcomes or those significantly differing between groups—such as bifurcation angle, bifurcation carina angle, plaque type, branching point to carina tip length, minimal lumen area at bifurcation, classification of bifurcation as true or non-true, SB ostium area, and the mean, minimal, and maximal diameter at the carina level in the main vessel (MV)—served as covariates for calculating the propensity score. The SB protection strategy was marked as the group indicator. For PSM, a 1:1 nearest neighbor approach with a caliper of 0.2 standard deviation of logit was used. The analysis was performed using SPSS 25.0 (IBM, Chicago, IL, USA). A two-tailed *p*-value of <0.05 was considered statistically significant.

## 3. Results

### 3.1 Patient Baseline Characteristics

**Fig. 1.**
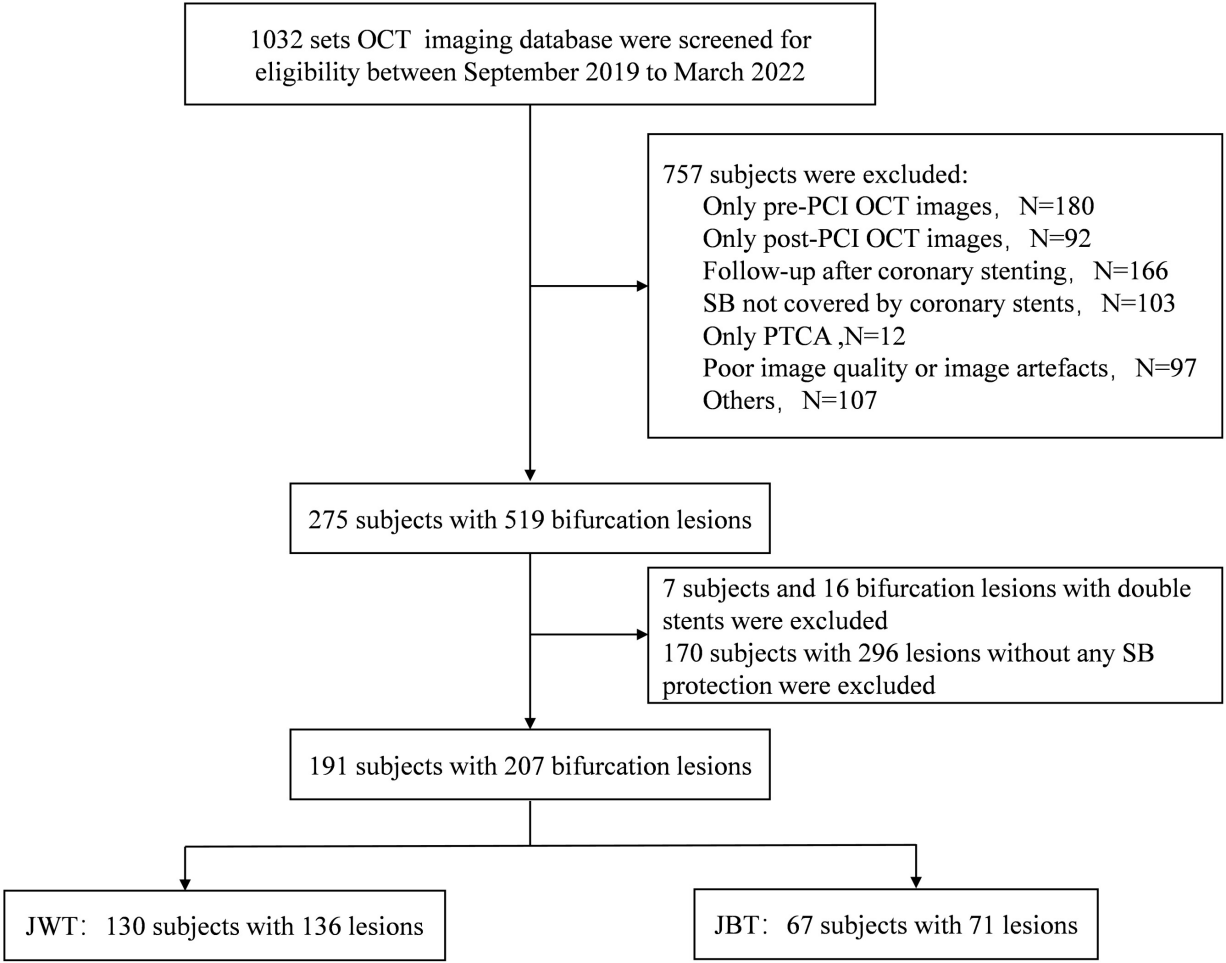
**Patient selection and lesion treatment overview in bifurcation lesions**. This figure outlines the screening and inclusion process of patients undergoing PCI and OCT, detailing the distribution of bifurcation lesions treated with the JBT and JWT. Specifically, four patients with two bifurcation lesions were treated with JBT simultaneously, while three patients with two lesions were treated with JWT simultaneously. Additionally, three patients with two lesions were each treated with both JBT and JWT. In another case, one patient received JBT for one lesion and JWT for two others. OCT, optical coherence tomography; PCI, percutaneous coronary intervention; PTCA, percutaneous transluminal coronary angioplasty; SB, side branch; JBT, jailed balloon technique; JWT, jailed wire technique.

In the current study, a total of 1032 patients who underwent PCI and OCT examinations were initially screened for eligibility, out of which 841 were excluded due to various criteria. Consequently, the final dataset comprised 207 bifurcation lesions in 191 patients, forming the final dataset for investigation (Fig. 1).

### 3.2 Angiographic Characteristics

In the study, the prevalence of true bifurcation lesions was significantly elevated in the JBT treatment cohort when compared to the JWT treatment cohort (73.2% vs. 47.1%, *p* < 0.001). Despite this, both groups showed comparable bifurcation and carina angles. Notably, before PCI, the SB ostium area in the JBT group was smaller than in the JWT group (2.40 [1.69, 3.26] vs. 3.09 [1.81, 4.55], *p* = 0.002, Table 1).

**Table 1.** **Baseline and angiographic characteristics**.

Characteristics		JWT	JBT	*p*
Bifurcation location, n (%)				0.535
	Left main	22 (16.2)	10 (14.1)	
	LAD diagonal	96 (70.6)	56 (78.9)	
	LCX-OM	14 (10.3)	4 (5.6)	
	RCA-PDA	4 (2.9)	1 (1.4)	
Medina classification, n (%)				0.000
	True bifurcation	64 (47.1)	52 (73.2)	
	0, 1, 1	13 (9.6)	11 (15.5)	
	1, 0, 1	7 (5.1)	2 (2.8)	
	1, 1, 1	44 (32.4)	39 (54.9)	
	Non-true bifurcation	72 (52.9)	19 (26.8)	
	0, 0, 1	2 (1.5)	3 (4.2)	
	1, 1, 0	17 (12.5)	6 (8.5)	
	0, 1, 0	36 (26.5)	8 (11.3)	
	1, 0, 0	17 (12.5)	2 (2.8)	
Plaque type				0.040
	Normal	2 (1.5)	1 (1.4)	
	Fibrous plaques	66 (48.5)	20 (28.2)	
	Lipid-rich plaques	37 (27.2)	25 (35.2)	
	Fibrocalcific plaques	31 (22.8)	25 (35.2)	
Bifurcation angle (°)		52.50 (41.81, 69.38)	52.75 (35.57, 63.58)	0.256
Bifurcation carina angle (°)		53.72 (34.08, 75.75)	54.29 (32.90, 71.16)	0.607
Minimum diameter of bifurcation (mm)		1.90 (1.54, 2.40)	1.67 (1.44, 2.00)	0.011
Maximum diameter of bifurcation (mm)		2.52 (2.17, 3.08)	2.31 (1.90, 2.61)	0.007
Mean diameter of bifurcation (mm)		2.24 (1.93, 2.79)	2.15 (1.76, 2.37)	0.026
MV area of bifurcation (mm^2^)		3.74 (2.69, 5.69)	3.23 (2.20, 4.26)	0.009
MLA in bifurcation (mm^2^)		1.72 (1.27, 2.25)	1.52 (1.09, 2.10)	0.076
Branching point- carina tip length (mm)		1.60 (1.10, 2.08)	1.40 (1.20, 1.80)	0.177
SB ostium area pre-PCI (mm^2^)		3.09 (1.81, 4.55)	2.40 (1.69, 3.26)	0.002

All values are presented as n (%), mean ± SD or median (interquartile range). JWT, jailed wire technique; JBT, jailed balloon technique; LAD, left anterior descending artery; LCX, left circumflex artery; OM, obtuse marginal branch; RCA, right coronary artery; PDA, right posterior descending artery; SB, side branch; MV, main vessel; MLA, minimal lumen area; PCI, percutaneous coronary intervention; SD, standard deviation.

### 3.3 The Procedural of A-JBT

The JBT was utilized in 71 bifurcation lesions, with active JBT (A-JBT) employed in 62 cases. The balloons in the procedures had a mean diameter of 2.01 ± 0.31 mm, and a mean dilation pressure of 7.65 ± 3.13 atmospheres (atm). Notably, in every case, the ratio of the diameter of the jailed balloon to the diameter of the side branch was maintained at less than 1:1.

### 3.4 Procedure Related Complications

The application of this technique resulted in successful outcomes in all patients. Notably, there were no cases of entrapment or fracture of the guidewire in either group, nor were there any incidents of jailed balloon entrapment in the JBT group. However, a single case of type B coronary artery dissection, as determined by the National Heart, Lung, and Blood Institute (NHLBI) criteria, was observed in the JBT group [18]. Based on the operator’s discretion, no further interventions were performed for this patient as they did not exhibit significant ischemic symptoms and there was no progression in the dissection’s severity.

### 3.5 The SB Ostium Area Difference between JBT and JWT

**Fig. 2.**
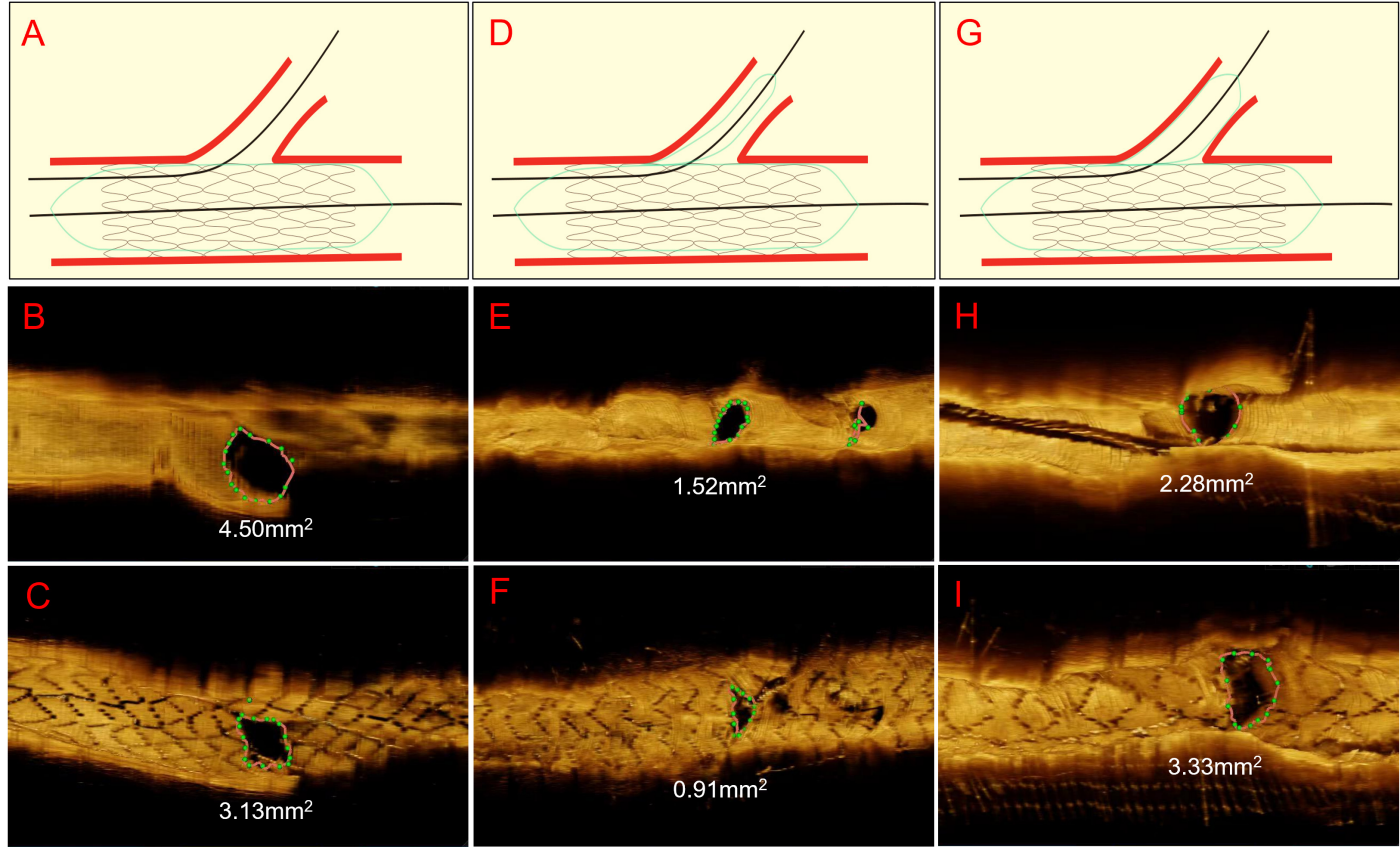
**Representative cases of OCT imaging pre- and post-PCI**. This figure presents a visual comparison of the SB ostium area before and after stent placement in coronary artery bifurcation lesions, as captured through OCT imaging. The figure is divided into three main sections, each depicting a different stenting technique: JWT, C-JBT, and A-JBT. The first section (A,B,C) includes a schematic of the JWT, followed by OCT images showcasing the SB ostium area (as green dotted lines) before (B) and after (C) PCI. In this JWT case, the area of the SB ostium decreases from 4.5 ${\mathrm{mm}}^{2}$ pre-PCI to 3.13 ${\mathrm{mm}}^{2}$ post-PCI. The second section (D,E,F) illustrates the C-JBT approach. Similarly, it begins with a schematic drawing (D), with pre-PCI (E) and post-PCI (F) OCT images showing a reduction in the SB ostium area from 1.52 ${\mathrm{mm}}^{2}$ to 0.91 ${\mathrm{mm}}^{2}$, indicating a decrease after stenting. The final section (G,H,I) represents the A-JBT, starting with its schematic (G) and OCT images before (H) and after (I) PCI. Unlike the other techniques, the A-JBT results in an increase in the SB ostium area from 2.28 ${\mathrm{mm}}^{2}$ before PCI to 3.33 ${\mathrm{mm}}^{2}$ afterward, showcasing its effectiveness in preserving or enhancing the SB ostium area. This figure demonstrates the differential impacts of JWT, C-JBT, and A-JBT on the SB ostium area, highlighting the potential advantage of A-JBT in maintaining or improving this critical region following stenting. OCT, optical coherence tomography; SB, side branch; PCI, percutaneous coronary intervention; JWT, jailed wire technique; C-JBT, conventional jailed balloon technique; A-JBT, active jailed balloon technique.

We determined the JBT group experienced a significantly greater SB ostium area increase compared to the JWT group (0.41 ± 1.22 mm^2^ vs. –0.25 ± 1.40 mm^2^, *p* = 0.001) (Table 2). Furthermore, analysis of true and non-true bifurcation subgroups between JWT and JBT also revealed significant increases to the SB ostium area. For true bifurcation the JBT group had a value of 0.38 ± 1.17 mm^2^ vs. –0.10 ± 1.10 mm^2^, for the JBT group (*p* = 0.023, Table 2). The non-true bifurcation subgroup had a JBT value of 0.49 ± 1.36 mm^2^ while the JBT had a value of –0.38 ± 1.62 mm^2^ (*p* = 0.034). Further analysis revealed the SB protective effect seen in JBT, when compared to JWT, could be attributed to A-JBT. The improvement in SB ostium area with A-JBT was consistent across lesion types, with significant increases seen in total (0.50 ± 1.27 mm^2^ vs. –0.25 ± 1.40 mm^2^, *p* = 0.001), true bifurcation lesions (0.47 ± 1.22 mm^2^ vs. –0.10 ± 1.10 mm^2^, *p* = 0.011) and non-true bifurcation lesions (0.56 ± 1.43 mm^2^ vs. –0.38 ± 1.62 mm^2^, *p* = 0.030). However, when comparing JWT to C-JBT, no significant differences were observed in the change in SB ostium area across all, true, and non-true bifurcation lesions, indicating a distinct advantage of A-JBT in SB protection (Table 2). Representative OCT images for JWT, C-JBT, A-JBT before and after PCI are illustrated in Fig. 2.

**Table 2.** **SB ostium area difference between the subgroups**.

	SB ostium area difference (mm^2^)								
Groups	JWT	JBT	*p*	JWT	A-JBT	*p*	JWT	C-JBT	*p*
Total	–0.25 ± 1.40	0.41 ± 1.22	0.001	–0.25 ± 1.40	0.50 ± 1.27	0.001	–0.25 ± 1.40	–0.17 ± 0.52	0.862
True bifurcation	–0.10 ± 1.10	0.38 ± 1.17	0.023	–0.10 ± 1.10	0.47 ± 1.22	0.011	–0.10 ± 1.10	–0.18 ± 0.59	0.859
Non-true bifurcation	–0.38 ± 1.62	0.49 ± 1.36	0.034	–0.38 ± 1.62	0.56 ± 1.43	0.030	–0.38 ± 1.62	–0.14 ± 0.34	0.833

Note: Lesion level. All values are presented as the mean ± SD. SB, side branch; JWT, jailed wire technique; JBT, jailed balloon technique; A-JBT, active jailed balloon technique; C-JBT, conventional jailed balloon technique; SD, standard deviation.

After propensity score matching (PSM), to equalize the coronary vascular structural factors between groups, no significant difference in SB ostium protection was observed between JBT and JWT, although JBT showed a non-significant trend towards better outcomes (0.28 ± 1.06 mm^2^ vs. –0.02 ± 1.29 mm^2^, *p* = 0.165) (**Supplementary Table 1**).

### 3.6 The Protection Role of Jailed Balloon Diameter and Pressure

Assessing the relationship between jailed balloon characteristics and the resulting changes to the SB ostium area led to the following findings. The correlation coefficient between jailed balloon pressure and the change in SB ostium area was 0.118 (*p* = 0.327), indicating a lack of significant correlation. Conversely, a significant correlation was found between the jailed balloon diameter and the SB ostium area difference, with a coefficient of 0.307 (*p* = 0.009) (**Supplementary Fig. 1**). There was a significant positive partial correlation between the diameter of the jailed balloon and the SB ostium area difference (r = 0.296, *p* = 0.013) suggesting that larger balloon diameters are associated with greater increases in the SB ostium area. However, no significant correlation was observed between the jailed balloon pressure and the SB ostium area difference (r = 0.083, *p* = 0.495).

After dividing the A-JBT group into two subgroups based on the dilation pressure (≤4 atm vs. >4 atm), we observed no significant difference in the SB ostium area across the three lesion categories, including total, true bifurcation lesions, and non-true bifurcation lesions. However, when the group was divided based on the jailed balloon diameter (≤2.0 mm vs. >2.0 mm), the SB ostium area difference in the large diameter subgroup was found to be significantly different, with greater areas seen in all of bifurcation lesions compared to the small diameter subgroup (1.44 ± 1.34 mm^2^ vs. 0.22 ± 1.10 mm^2^, *p* = 0.002) (Table 3). These findings suggest that the physical dimensions of the jailed balloon, particularly its diameter, play a more pivotal role in influencing the post-procedural SB ostium area than the applied pressure during dilation.

**Table 3.** **SB ostium area difference between jailed balloon dilation pressure and diameter**.

	SB ostium area difference (mm^2^)					
Groups	Low pressure (≤4 atm)	High pressure (>4 atm)	*p*	Small diameter (≤2.0 mm)	Large diameter (>2.0 mm)	*p*
Total	0.59 ± 0.95	0.46 ± 1.37	0.713	0.22 ± 1.10	1.44 ± 1.34	0.002
True bifurcation	0.32 ± 0.65	0.52 ± 1.35	0.639	0.30 ± 1.13	1.74 ± 1.02	0.037
Non-true bifurcation	1.10 ± 1.25	0.36 ± 1.54	0.267	–0.11 ± 0.93	1.32 ± 1.48	0.019

Note: Lesion level. All values are presented as the mean ± SD. SB, side branch; SD, standard deviation.

## 4. Discussion

To the best of our knowledge, this study is the first to compare the protective effect of the JBT and the JWT in bifurcation lesions using 3D-OCT. The key findings of this study can be summarized as follows: (1) The protective effect of JBT primarily stems from the impact of A-JBT. (2) The larger diameter of the jailed balloon, rather than the higher pressure, provides greater protection for the SB.

While SB ostium stenosis may worsen due to plaque or carina shift following MV stenting, adopting a single stent technique with SB stenting is still the recommended approach for treating coronary bifurcation lesions [19]. This clinical approach is justified by evidence showing that a smaller SB ostium area is associated with decreased fractional flow reserve values [20]. This correlation suggests that compromised SB integrity is a significant concern, and is likely to result in adverse clinical outcomes in PCI bifurcation [20]. Consequently, considerable efforts have been made to identify patients at risk for SB compromise following MV stent implantation [6, 8, 10, 21, 22, 23, 24]. Although the JBT and JWT techniques are established methods for preventing SB occlusion during bifurcation lesion treatment, their effectiveness in protecting against SB occlusion remains controversial [10, 11, 12, 13]. Previous studies have suggested that JBT is more effective than JWT in preventing SB occlusion, but these studies relied on visual assessment of coronary angiography or quantitative coronary angiography [8, 12]. However, visual estimation based on angiography or quantitative coronary angiography is not reliable for assessing the severity of SB lesions [14, 25]. Therefore, more accurate tools are needed to measure the extent of SB ostial stenosis.

As a state-of-the-art imaging modality, OCT plays a critical role in measuring the side branch ostium [16]. The accuracy of SB ostial area measurements using 3D-OCT in MV closely matches that of SB OCT pullback, suggesting that 3D-OCT guidance for optimal SB treatment is a viable and effective solution [15, 26]. Therefore, this study aimed to evaluate the impact of JBT versus JWT on the SB ostial area in bifurcation lesions treated with the single-stent approach. When considering all bifurcation lesions, JBT was associated with a relatively larger absolute SB ostium area compared to JWT. In the subgroup analysis, JBT demonstrated a superior protective effect, primarily attributed to A-JBT. In contrast, C-JBT did not show any discernible advantage over JWT. Previous studies have shown that A-JBT can prevent SB occlusion, and even improve SB functional blood flow compared to JWT [11, 27, 28]. This superiority of JBT over JWT was consistent across both true and non-true bifurcation lesion subgroups. Furthermore, PSM analysis was utilized to compensate for potential selection bias in retrospective observational studies and to facilitate an intention-to-treat analysis. Although no statistical difference was observed, there was a trend suggesting that the increase in SB ostium area may be greater in the JBT group compared to the JWT group.

Carina and plaque shifts are the primary mechanisms leading to SB ostial compromise at the SB following MV stent implantation [29]. The protective effect of JBT on SB ostium may be attributed to its capacity to mitigate these shifts. We have demonstrated that the utilization of a large diameter jailed balloon and A-JBT provide superior protection in true bifurcation lesions. Subgroup analysis revealed that a larger jailed balloon diameter (>2.0 mm) produced greater SB ostium area differences when compared to a smaller diameter (≤2.0 mm). Despite these insights, there is a notable gap in research directly comparing jailed balloon diameter and SB protection, necessitating additional studies to confirm our findings. A previous study found no difference in the level of dilation pressure of the jailed balloon and its protective effect on SB [30], which was confirmed by our results.

Several studies have assessed the protective effect of JBT on SB through coronary angiography, finding JBT superior to JWT in reducing branch occlusion, but not in long-term patient outcomes [11, 12]. Our study aligns with these findings to an extent, confirming that JBT, particularly A-JBT, offers more effective protection of the SB ostium area compared to JWT. Nonetheless, the difference in the increase of the SB ostium area between the JBT and JWT groups was modest, at an average of 0.66 mm^2^ in the JBT group compared to the JWT group (0.41 mm^2^ vs. –0.25 mm^2^). This relatively small disparity in SB ostium area enlargement may explain the absence of observed significant differences in the long-term prognosis of patients treated with either technique [11].

The JBT group was characterized by a smaller initial SB ostium area and a greater incidence of true bifurcation cases, which aligns with observations from clinical practice. Despite the strategic use of JBT, the extent of SB ostium enlargement following PCI in the JBT group was modest, with a mean difference of 0.41 mm^2^. This finding suggests that A-JBT, particularly when utilizing larger diameter balloons, may be more suitable for treating relatively large SBs with ostial stenosis [31]. Such scenarios include bifurcations of the non-left main trunk and the left main trunk when associated with a smaller left circumflex artery [31]. However, the implementation of A-JBT necessitates a careful consideration of potential risks, including SB dissection, the entrapment of devices, and stent deformation, which could complicate the procedure [32, 33]. In our study, balloons ranging from 1.5–2.5 mm were used for SB treatment via JBT, and no significant complications were reported, suggesting a cautious yet effective application for this technique.

## 5. Limitations

This study has several limitations that merit acknowledgment. Firstly, its retrospective design inherently carries the risk of introducing various certain biases into the findings. Secondly, the selection of patients and procedural techniques was subject to the operating clinician’s subjective preferences, potentially leading to selection bias. However, it’s noteworthy that the basic demographic and clinical characteristics, such as age, sex, and clinical diagnosis were similar across the groups, somewhat mitigating concerns related to selection bias. Thirdly, the initial SB ostium area was smaller in the JBT group compared to the JWT group before undergoing PCI. To counteract these confounding factors, PSM was employed, aiming to equalize these variables and reduce the impact of biases on the study’s outcomes.

## 6. Conclusions

Based on the analysis of the series OCT imaging database, A-JBT was found to be superior to JWT in preserving SB ostial area in single stent procedures. Notably, the efficacy of protection afforded to the SB was more strongly associated with the diameter of the jailed balloon than with the pressure applied during dilation. Therefore, when using the provisional stenting technique for bifurcation lesions, utilizing a larger diameter balloon and ensuring its protection with A-JBT appears to be a preferable strategy for SB preservation. Nonetheless, the validation of these findings necessitates further investigation through prospective studies and randomized clinical trials to solidify the evidence base supporting this approach.

## Data Availability

The data that support the findings of this study are available from the corresponding author upon reasonable request.

## Abbreviations

OCT, optical coherence tomography; SB, side branch; MV, main vessel; 3D, three-dimensional; JWT, jailed wire technique; JBT, jailed balloon technique; PCI, percutaneous coronary intervention; PSM, propensity score matching.

## Supplementary Material

Supplementary material associated with this article can be found, in the online version, at https://doi.org/10.31083/j.rcm2508300.
