# Supplementary material for: Jailed Balloon Technique Versus Jailed Wire Technique for Side Branch Ostium Protection in Bifurcation Lesions: Evidence from Three-dimensional Optical Coherence Tomography Analysis
Source: Rev Cardiovasc Med. 2024 Aug 21;25(8):300. doi: 10.31083/j.rcm2508300 (PMC11366982; doi:10.31083/j.rcm2508300)
Supplement: Supplementary file 1 [file 2153-8174-25-8-300-s1.docx]

**Supplementary**

**Table S1. Angiographic characteristics and SB ostium area difference between the subgroups after PSM**

| Characteristics | JWT (60) | JBT (60) | *P* |
| --- | --- | --- | --- |
| True bifurcation | 43(71.7) | 41(68.3) | 0.690 |
| Plaque type in MV |  |  | 0.238 |
| Normal | 0(0) | 1(1.7) |  |
| Fibrous plaques | 25(41.7) | 19(31.7) |  |
| Lipid-rich plaques | 14(23.3) | 22(36.7) |  |
| Fibrocalcific plaques | 21(35.0) | 18(30.0) |  |
| Bifurcation angle(°) | 51.25(41.54,67.34) | 52.82(36.53,63.44) | 0.637 |
| Bifurcation carina angle(°) | 53.72(30.62,79.60) | 54.00(33.03,68.27) | 0.832 |
| Minimum diameter of bifurcation (mm) | 1.71(1.48,2.07) | 1.67(1.44,2.04) | 0.548 |
| Maximum diameter of bifurcation (mm) | 2.42±0.61 | 2.41±0.56 | 0.895 |
| Mean diameter of bifurcation (mm) | 2.20±0.56 | 2.20±0.52 | 0.964 |
| Branching point- carina tip length(mm) | 1.30(1.00,2.00) | 1.5(1.23,1.80) | 0.264 |
| MV area of bifurcation (mm^2^) | 3.19(2.37,4.52) | 3.41(2.29,4.32) | 0.844 |
| MLA in bifurcation (mm^2^) | 1.68±0.71 | 1.72±0.77 | 0.783 |
| SB ostium area pre-PCI (mm^2^) | 2.03(1.57,3.09) | 2.51(1.82,3.42) | 0.141 |
| SB ostium area difference (mm^2^) | -0.02±1.29 | 0.28±1.06 | 0.165 |

Note: Lesion level

All values are presented as the mean ± SD or median (interquartile range). SB, side branch; JWT, jailed wire technique; JBT, jailed balloon technique; PSM, propensity score matching; MV, main vessel; MLA, minimal lumen area; PCI, percutaneous coronary intervention.

**Figure S1. Associations between the SB ostium area difference and jailed balloon diameter and dilation pressure**


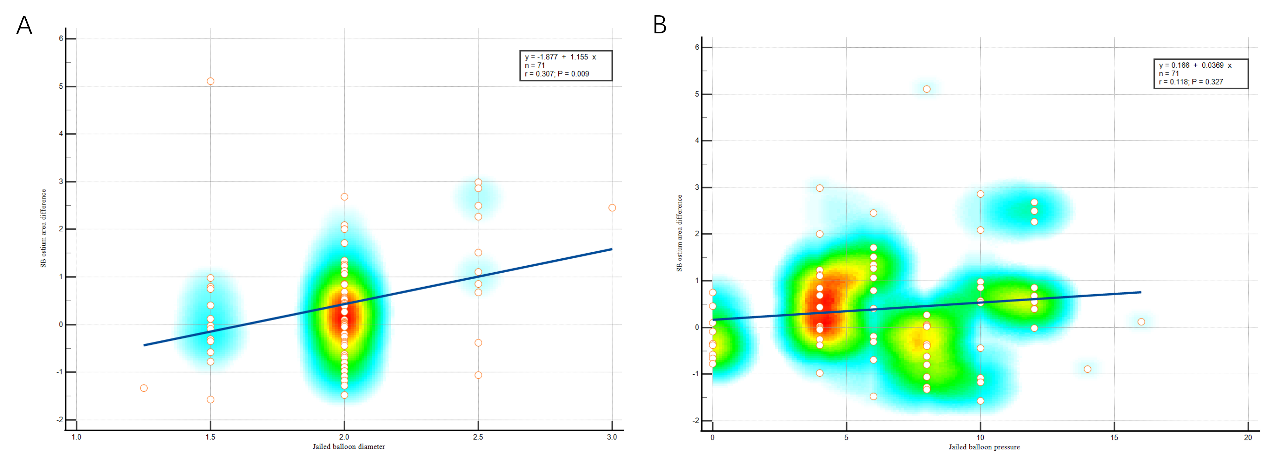


The SB ostium area difference was significantly associated with large jailed balloon diameter, r=0.307, P=0.009 (A) but not with jailed balloon dilation pressure, r=0.118, P=0.327 (B).
